# Inpatient Administration of Alpha-1-Adrenergic Receptor Blocking Agents Reduces Mortality in Male COVID-19 Patients

**DOI:** 10.3389/fmed.2022.849222

**Published:** 2022-02-28

**Authors:** Shilong Li, Tomi Jun, Jonathan Tyler, Emilio Schadt, Yu-Han Kao, Zichen Wang, Maximilian F. Konig, Chetan Bettegowda, Joshua T. Vogelstein, Nickolas Papadopoulos, Ramon E. Parsons, Rong Chen, Eric E. Schadt, Li Li, William K. Oh

**Affiliations:** ^1^Sema4, Stamford, CT, United States; ^2^Lustgarten Laboratory, Ludwig Center, The Howard Hughes Medical Institute, The Johns Hopkins Kimmel Cancer Center, Baltimore, MD, United States; ^3^Division of Rheumatology, Department of Medicine, The Johns Hopkins University School of Medicine, Baltimore, MD, United States; ^4^Department of Neurosurgery, The Johns Hopkins University School of Medicine, Baltimore, MD, United States; ^5^Department of Biomedical Engineering, Institute for Computational Medicine, The Johns Hopkins University, Baltimore, MD, United States; ^6^Department of Biostatistics, Bloomberg School of Public Health, The Johns Hopkins University, Baltimore, MD, United States; ^7^Department of Oncology and Pathology, The Johns Hopkins University School of Medicine, Baltimore, MD, United States; ^8^Division of Hematology and Medical Oncology, Icahn School of Medicine at Mount Sinai, New York, NY, United States; ^9^Tisch Cancer Institute, Icahn School of Medicine at Mount Sinai, New York, NY, United States; ^10^Department of Genetics and Genomic Sciences, The Icahn Institute for Genomics and Multiscale Biology, Icahn School of Medicine at Mount Sinai, New York, NY, United States

**Keywords:** off-label drug use, alpha-1-adrenergic receptor antagonist, coronavirus disease, infectious disease, multivariate logistic analysis, real-world evidence, electronic medical record, COVID-19

## Abstract

Apha-1-adrenergic receptor antagonists (α_1_-blockers) can suppress pro-inflammatory cytokines, thereby potentially improving outcomes among patients with COVID-19. Accordingly, we evaluated the association between α_1_-blocker exposure (before or during hospitalization) and COVID-19 in-hospital mortality. We identified 2,627 men aged 45 or older who were admitted to Mount Sinai hospitals with COVID-19 between February 24 and May 31, 2020, in New York. Men exposed to α_1_-blockers (*N* = 436) were older (median age 73 vs. 64 years, *P* < 0.001) and more likely to have comorbidities than unexposed men (*N* = 2,191). Overall, 777 (29.6%) patients died in hospital, and 1,850 (70.4%) were discharged. Notably, we found that α_1_-blocker exposure was independently associated with improved in-hospital mortality in a multivariable logistic analysis (OR 0.699; 95% CI, 0.498-0.982; *P* = 0.039) after adjusting for patient demographics, comorbidities, and baseline vitals and labs. The protective effect of α_1_-blockers was stronger among patients with documented inpatient exposure to α_1_-blockers (OR 0.624; 95% CI 0.431-0.903; *P* = 0.012). Finally, age-stratified analyses suggested variable benefit from inpatient α_1_-blocker across age groups: Age 45-65 OR 0.483, 95% CI 0.216-1.081 (*P* = 0.077); Age 55-75 OR 0.535, 95% CI 0.323-0.885 (*P* = 0.015); Age 65-89 OR 0.727, 95% CI 0.484-1.092 (*P* = 0.124). Taken together, clinical trials to assess the therapeutic value of α_1_-blockers for COVID-19 complications are warranted.

## Introduction

Severe coronavirus disease 2019 (COVID-19) has been linked to dysregulated immune responses, including an excessive inflammatory response marked by high levels of proinflammatory cytokines such as interleukin-6 (IL-6) (1, 2). Immunosuppressive drugs such as glucocorticoids have become a standard component of treatment for severe COVID-19 (3), and several trials of anti-cytokine or anti-inflammatory agents are underway or have reported promising results (4–10). Despite these advances, there remains a need for safe, effective, and widely available therapeutic options.

Adrenergic signaling has been linked to hyperinflammation in models of bacterial sepsis and cytokine release syndrome. In preclinical experiments, a positive feedback loop of adrenergic signaling was identified wherein macrophages responded to catecholamines by producing more catecholamines and inflammatory cytokines; this adrenergic loop could be interrupted by blocking α_1_-adrenergic receptors with prazosin (11). In a retrospective clinical study of patients with acute respiratory distress and pneumonia, exposure to α_1_-adrenergic receptor antagonists (α_1_-blockers) was associated with a significant reduction in risk of mechanical ventilation or death (12). Similarly, a recent retrospective analysis of 25,130 patients with COVID-19 across the United States Veterans Health Administration hospital system showed that outpatient exposure to any α_1_-blocker was associated with decreased in-hospital mortality compared to matched controls not on any α_1_-blocker at the time of hospital admission (13).

These observations have led to the hypothesis that α_1_-blockers in routine clinical use (e.g., prazosin, doxazosin, tamsulosin, etc.) may be repurposed for COVID-19 treatment (14). We conducted this real-world evidence study based on electronic medical record (EMR) data to determine whether exposure to α_1_-blockers is independently associated with mortality among patients hospitalized with COVID-19.

## Results

### Patient Characteristics and Outcomes

**Figure 1:**
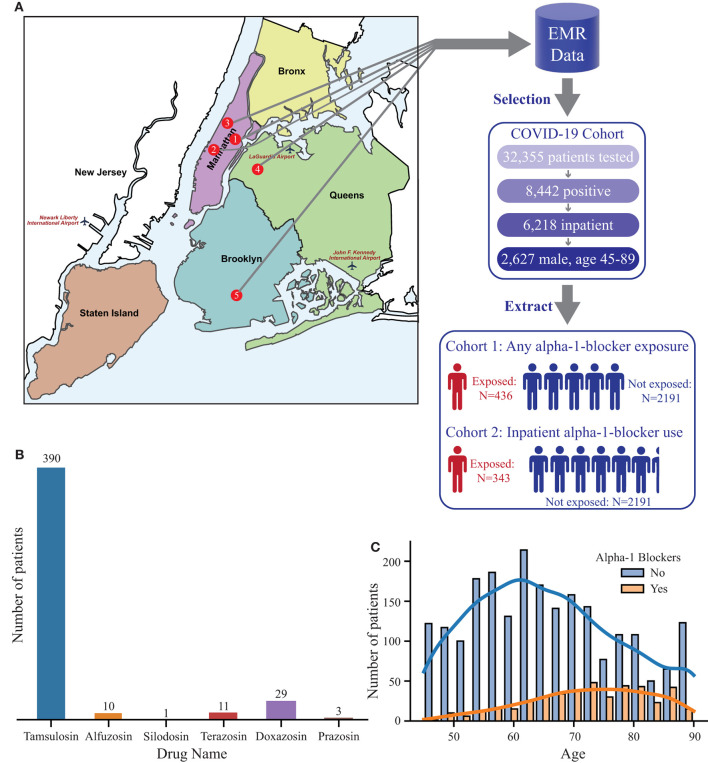
Study design and overview. **(A)** Schematic flowchart of data extraction and analysis plan. Data was collected from five Mount Sinai hospitals: 1) The Mount Sinai Hospital, 2) Mount Sinai West, 3) Mount Sinai St. Luke's, 4) Mount Sinai Queens, and 5) Mount Sinai Brooklyn. A total of 32,355 patients were tested, of which 2,627 were positive, inpatient, and male ages 45-89. Data was extracted for the 2,627 male, age 45-89 patients and split into two cohorts: 1) any alpha-1-blocker exposure and those not exposed and 2) inpatient alpha-1-blocker exposure and those not exposed. The image of the boroughs of NYC was adapted from https://commons.wikimedia.org/wiki/File:New_York_City_District_Map.svg, which is under a Creative Commons Attribution-Share Alike 2.5 Generic license. **(B)** Prevalence of various alpha-1-blockers in the exposed cohort. **(C)** Age distribution of those exposed to alpha-1-blockers (red) and those not exposed (blue).

We gathered and processed data from five hospitals within the Mount Sinai Health System to construct three cohorts of male patients aged 45 years or older: (a) no α_1_-blocker exposure (*N* = 2,191), (b) an α_1_-blocker-exposed group (*N* = 436), and (c) a documented inpatient α_1_-blocker-exposed group (*N* = 343) (Figure 1A). The most common α_1_-blocker was Tamsulosin followed by Doxazosin (Figure 1B). See Materials and Methods for details regarding data processing and cohort generation.

The α_1_-blocker exposed group was older (median age 73 vs. 64 years, *P* < 0.001) and more likely to have comorbidities than the unexposed group (Figure 1C; Table 1). Chronic diseases such as COPD, hypertension, diabetes mellitus, chronic kidney disease, cancer, and cardiovascular disease were significantly enriched in the α_1_-blocker group. In addition, the α_1_-blocker exposed group had more severe hypoxia during their hospitalizations (median oxygen saturation nadir: 88%, IQR 78-91 vs. 90%, IQR 82-94%, *P* < 0.001) and a higher rate of ICU admission (25.7 vs. 19.7%, *P* = 0.006). Other patient demographics are shown in Table 1.

**Table 1:** Baseline characteristics and hospitalization outcomes, by alpha-1-blocker exposure.

	**Non-alpha-blockers**	**Alpha-blockers**	***P*-value**	**Inpatient alpha-blockers**	***P*-value**
***N***	**2,191**	**436**		**343**	
**PATIENT CHARACTERISTICS**					
Age	64 [56,74]	73 [66,81]	<0.001	73 [65, 80]	<0.001
Race, *n* (%)			0.004		0.065
African-American	514 (23.5)	86 (19.7)		68 (19.8)	
Asian	114 (5.2)	19 (4.4)		12 (3.5)	
Hispanic	624 (28.5)	130 (29.8)		104 (30.3)	
Other	336 (15.3)	49 (11.2)		43 (12.5)	
Unknown	69 (3.1)	10 (2.3)		10 (2.9)	
White	534 (24.4)	142 (32.6)		106 (30.9)	
BMI (kg/m^2^)	27.0 [23.8,30.9]	26.0 [23.2,30.0]	0.003	26.5 [23.6, 30.2]	0.113
Smoking status, *n* (%)			<0.001		0.004
Never	1,023 (46.7)	202 (46.3)		158 (46.1)	
Not asked	525 (24.0)	66 (15.1)		60 (17.5)	
Quit	527 (24.1)	148 (33.9)		110 (32.1)	
Yes	116 (5.3)	20 (4.6)		15 (4.4)	
Temperature (F)	98.6 [97.9,99.8]	98.5 [98.0,99.5]	0.365	98.6 [98.0, 99.7]	0.827
Max. temperature (F)	100.4 [99.1,102.1]	100.8 [99.6,102.4]	<0.001	101.1 [99.8, 102.5]	<0.001
O2 Saturation (%)	96.0 [92.0,98.0]	95.0 [92.0,97.0]	0.168	95.0 [92.0, 97.0]	0.056
Min. O2 saturation (%),	90.0 [82.0,94.0]	88.0 [78.0,91.0]	<0.001	88.0 [77.0, 91.0]	<0.001
Heart rate (BPM)	94.0 [82.0,108.0]	91.0 [77.0,104.0]	0.001	90.0 [77.0, 103.0]	<0.001
Respiratory rate > 25, *n* (%)	317 (14.5)	64 (14.7)	0.968	52 (15.2)	0.798
High BP, *n* (%)	817 (37.3)	163 (37.4)	0.987	134 (39.1)	0.567
**DRUGS**					
ACE inhibitors, *n* (%)	267 (12.2)	90 (20.6)	<0.001	70 (20.4)	<0.001
ARBs, *n* (%)	222 (10.1)	88 (20.2)	<0.001	70 (20.4)	<0.001
Diuretics, *n* (%)	629 (28.7)	203 (46.6)	<0.001	155 (45.2)	<0.001
Beta blockers, *n* (%)	623 (28.4)	204 (46.8)	<0.001	154 (44.9)	<0.001
Calcium-channel blockers, *n* (%)	627 (28.6)	213 (48.9)	<0.001	161 (46.9)	<0.001
Statin, *n* (%)	711 (32.5)	249 (57.1)	<0.001	193 (56.3)	<0.001
Glucocorticoid, *n* (%)	745 (34.0)	218 (50.0)	<0.001	167 (48.7)	<0.001
**COMORBIDITIES**					
Asthma, *n* (%)	52 (2.4)	17 (3.9)	0.098	12 (3.5)	0.294
COPD, *n* (%)	76 (3.5)	30 (6.9)	0.002	19 (5.5)	0.085
Hypertension, *n* (%)	625 (28.5)	186 (42.7)	<0.001	142 (41.4)	<0.001
OSA, *n* (%)	36 (1.6)	14 (3.2)	0.046	13 (3.8)	0.013
Obesity, *n* (%)	127 (5.8)	29 (6.7)	0.563	23 (6.7)	0.589
Diabetes mellitus, *n* (%)	405 (18.5)	130 (29.8)	<0.001	99 (28.9)	<0.001
Chronic kidney disease, *n* (%)	216 (9.9)	75 (17.2)	<0.001	59 (17.2)	<0.001
HIV, *n* (%)	48 (2.2)	9 (2.1)	0.989	7 (2.0)	0.982
Cancer, *n* (%)	165 (7.5)	66 (15.1)	<0.001	45 (13.1)	0.001
Coronary artery disease, *n* (%)	255 (11.6)	90 (20.6)	<0.001	64 (18.7)	<0.001
Atrial fibrillation, *n* (%)	119 (5.4)	59 (13.5)	<0.001	37 (10.8)	<0.001
Heart failure, *n* (%)	131 (6.0)	51 (11.7)	<0.001	32 (9.3)	0.026
Chronic viral hepatitis, *n* (%)	27 (1.2)	7 (1.6)	0.691	4 (1.2)	1.00
Liver disease, *n* (%)	47 (2.1)	13 (3.0)	0.372	11 (3.2)	0.304
Acute kidney injury, *n* (%)	150 (6.8)	47 (10.8)	0.006	32 (9.3)	0.123
**BASELINE LABS**					
WBC (K/ul)	7.9 [5.7,10.9]	7.7 [5.4,11.4]	0.531	7.6 [5.4, 11.4]	0.527
Creatinine > 1.2, *n*(%)	903 (41.2)	231 (53.0)	<0.001	187 (54.5)	<0.001
Anion Gap (mEq/L)	12.3 [10.7,15.0]	12.4 [10.2,15.0]	0.413	12.4 [10.2, 14.7]	0.396
Potassium (mmol/L)	4.4 [4.0,4.8]	4.3 [3.9,4.8]	0.622	4.3 [3.9, 4.8]	0.200
ALT (U/L)	34.0 [21.0,57.0]	29.0 [18.0,50.0]	<0.001	30.0 [18.0, 51.0]	0.001
**HOSPITAL CONDITIONS**					
ICU, *n* (%)	431 (19.7)	112 (25.7)	0.006	94 (27.4)	0.001
Duration (days)	4.7 [1.1,9.5]	7.0 [3.6,11.5]	<0.001	7.5 [4.0, 12.0]	<0.001
Hospital status, *n* (%)			0.033		0.153
Deceased	629 (28.7)	148 (33.9)		112 (32.7)	
Discharged	1562 (71.3)	288 (66.1)		231 (67.3)	

*Patient characteristics are summarized as median and interquartile range (IQR) for continuous variables. Categorical variables are displayed as number and percentage (%). The Kruskal-Wallis test was used to test for differences for continuous variables, and the χ^2^ test was used for categorical variables*.

*BMI, body mass index; BPM, beats per minute; BP, blood pressure; SBP, systolic blood pressure; DBP, diastolic blood pressure; ACE, angiotensin-converting enzyme; ARBs, angiotensin II receptor blockers; COPD, chronic obstructive pulmonary disease; OSA, Obstructive Sleep Apnea; HIV, human immunodeficient virus; WBC, white blood cell count; ALT, alanine aminotransferase*.

Overall, 777 (29.6%) patients died, and 1,850 (70.4%) were discharged. Unadjusted in-hospital mortality was 33.9% in the exposed group and 28.7% in the unexposed group.

### α_1_-Blocker Exposure and COVID-19 In-Hospital Mortality

We evaluated the association of α_1_-blockers and outcomes of COVID-19 (death: *N* = 777; discharge: *N* = 1,850) using multivariate logistic regression models. In the overall population, α_1_-blocker exposure was significantly associated with a reduction in in-hospital mortality (OR 0.699; 95% CI, 0.498-0.982; *P* = 0.039) (Table 2). We report the unadjusted OR values in Supplementary Table 2.

**Table 2:** Multivariable logistic regression results for COVID-19 in-hospital mortality, by alpha-1-blocker exposure (any vs. inpatient use).

	**Overall cohort, any exposure** **(*****N*** **=** **2,627)**		**Overall cohort, inpatient use** **(*****N*** **=** **2,534)**	
	**Adjusted OR (95% CI)**	***P*-value**	**Adjusted OR (95% CI)**	***P*-value**
**DRUGS**				
Alpha-1 blockers	0.699 (0.498-0.982)	0.039	0.624 (0.431-0.903)	0.012
ACE inhibitors	1.119 (0.750-1.670)	0.583	1.020 (0.672-1.549)	0.925
ARBs	1.285 (0.852-1.937)	0.232	1.261 (0.821-1.937)	0.290
Diuretics	1.222 (0.879-1.698)	0.232	1.192 (0.848-1.675)	0.311
Beta blockers	1.496 (1.115-2.008)	0.007	1.581 (1.166-2.144)	0.003
Calcium-channel blockers	0.648 (0.476-0.883)	0.006	0.577 (0.418-0.796)	<0.001
Statin	0.830 (0.606-1.137)	0.246	0.831 (0.599-1.152)	0.267
Glucocorticoid	1.468 (1.070-2.015)	0.017	1.572 (1.134-2.178)	0.007
**PATIENT CHARACTERISTICS**				
Age	1.070 (1.054-1.086)	<0.001	1.069 (1.053-1.086)	<0.001
Race: African American	0.824 (0.545-1.246)	0.360	0.773 (0.505-1.183)	0.235
Race: Asian	0.836 (0.416-1.682)	0.616	0.733 (0.351-1.531)	0.409
Race: Hispanic	0.953 (0.656-1.384)	0.799	0.852 (0.579-1.254)	0.416
Race: Other	0.797 (0.508-1.250)	0.323	0.737 (0.465-1.168)	0.194
Race: Unknown	1.183 (0.493-2.836)	0.707	1.148 (0.473-2.788)	0.760
Smoking status: not asked	1.193 (0.820-1.735)	0.355	1.191 (0.812-1.746)	0.372
Smoking status: quit	1.439 (1.025-2.020)	0.036	1.387 (0.975-1.974)	0.069
Smoking status: yes	1.536 (0.789-2.990)	0.206	1.699 (0.864-3.340)	0.125
BMI	1.016 (0.994-1.038)	0.156	1.017 (0.995-1.040)	0.127
Temperature	0.988 (0.927-1.052)	0.697	0.983 (0.923-1.047)	0.598
Max. temperature	1.225 (1.126-1.333)	<0.001	1.223 (1.122-1.334)	<0.001
O2 saturation	1.000 (0.980-1.020)	0.997	1.003 (0.983-1.024)	0.777
Min. O2 saturation	0.946 (0.935-0.957)	<0.001	0.942 (0.930-0.954)	<0.001
Heart rate	0.995 (0.987-1.002)	0.158	0.993 (0.985-1.001)	0.082
Respiratory rate > 25	1.500 (1.053-2.137)	0.025	1.437 (0.997-2.072)	0.052
High BP (SBP > 140 or DBP > 90)	1.234 (0.925-1.645)	0.153	1.222 (0.907-1.646)	0.188
**COMORBIDITIES**				
Asthma	0.399 (0.156-1.020)	0.055	0.391 (0.142-1.074)	0.069
COPD	1.329 (0.717-2.463)	0.367	1.171 (0.610-2.249)	0.635
Hypertension	0.869 (0.608-1.243)	0.442	0.877 (0.603-1.275)	0.492
Obstructive sleep apnea	1.355 (0.549-3.346)	0.510	1.394 (0.552-3.519)	0.482
Obesity	1.245 (0.692-2.242)	0.465	1.248 (0.677-2.301)	0.478
Diabetes mellitus	0.940 (0.648-1.364)	0.745	0.939 (0.637-1.383)	0.749
Chronic kidney disease	1.184 (0.744-1.884)	0.476	1.236 (0.762-2.003)	0.391
HIV	1.930 (0.771-4.830)	0.160	1.479 (0.546-4.006)	0.442
Cancer	0.878 (0.558-1.382)	0.575	0.891 (0.554-1.435)	0.636
Coronary artery disease	1.007 (0.656-1.547)	0.973	1.106 (0.709-1.727)	0.657
Atrial fibrillation	0.903 (0.535-1.523)	0.701	0.947 (0.546-1.641)	0.846
Heart failure	0.789 (0.457-1.364)	0.397	0.866 (0.490-1.530)	0.620
Chronic viral hepatitis	0.672 (0.191-2.362)	0.536	0.535 (0.135-2.131)	0.375
Liver disease	1.244 (0.521-2.968)	0.623	1.494 (0.616-3.619)	0.374
Acute kidney injury	0.893 (0.539-1.480)	0.660	0.819 (0.477-1.405)	0.469
**LABS**				
WBC	1.018 (0.992-1.044)	0.175	1.022 (0.994-1.052)	0.126
Creatinine > 1.2	1.474 (1.062-2.047)	0.020	1.458 (1.038-2.049)	0.030
Anion gap	1.059 (1.023-1.096)	0.001	1.061 (1.024-1.100)	0.001
Potassium	1.157 (0.969-1.381)	0.106	1.103 (0.917-1.326)	0.299
ALT	1.001 (0.999-1.003)	0.440	1.001 (0.999-1.003)	0.488
Ferritin	1.000 (1.000-1.000)	0.886	1.000 (1.000-1.000)	0.836
**HOSPITAL CONDITIONS**				
ICU	2.617 (1.838-3.726)	<0.001	2.648 (1.839-3.812)	<0.001
Duration (days)	0.957 (0.937-0.978)	<0.001	0.959 (0.937-0.980)	<0.001

*ACE, angiotensin-converting enzyme; ARBs, angiotensin II receptor blockers; BMI, body mass index; BP, blood pressure; SBP, systolic blood pressure; DBP, diastolic blood pressure; COPD, chronic obstructive pulmonary disease; HIV, human immunodeficient virus; WBC, white blood cell count; ALT, alanine aminotransferase*.

We further assessed the impact of α_1_-blockers on mortality for patients with documented administration of α_1_-blockers while admitted to the hospital (*N* = 343) compared to unexposed patients. We observed that inpatient α_1_-blocker use significantly reduced the risk of in-hospital mortality overall (OR 0.624; 95% CI 0.431-0.903; *P* = 0.012) (Table 2).

Additionally, we investigated the impact of other medications on mortality using the same multivariate logistic-regression models. Notably, both beta-blockers (OR 1.496; 95% CI, 1.115-2.008; *P* = 0.007) and glucocorticoids (OR 1.468; 95% CI, 1.070-2.015; *P* = 0.017) were associated with increased mortality in the any exposure and the inpatient exposure cohorts. Conversely, calcium-channel blockers exhibited a significant reduction in mortality in the both the overall (OR 0.648; 95% CI, 0.476-0.883; *P* = 0.006) and inpatient exposure cohort (OR 0.577; 95% CI, 0.418-0.796; *P* < 0.001).

### Age-Stratified Associations of α_1_-Blockers and COVID-19 In-Hospital Mortality

To identify differences in the treatment effect of α_1_-blockers on different age groups, we segmented the population into three age groups (45-65; 55-75; 65-89) and analyzed each group separately using logistic regression, adjusting for the same covariates as the unstratified analysis. The age groups were overlapped by 10 years to preserve the sample size. Inpatient α_1_-blocker use was associated with a significantly lower risk of in-hospital mortality in the 55-75 age group (OR 0.535; 95% CI 0.323-0.885; *P* = 0.015), but not the 45-65 (OR 0.483; 95% CI 0.216-1.081; *P* = 0.077) and 65-89 age groups (OR 0.727; 95% CI 0.484-1.092; *P* = 0.124) (Table 3).

**Table 3:** Age-stratified multivariable logistic regression analysis for COVID-19 in-hospital mortality, by alpha-1-blocker exposure (any vs. inpatient use).

	**Overall cohort, any exposure**		**Overall cohort, inpatient use**	
	**Adjusted OR (95% CI)**	***P*-value**	**Adjusted OR (95% CI)**	***P*-value**
**Ages**				
45-65 (*N* = 1,159)	0.555 (0.266-1.163)	0.119	0.483 (0.216-1.081)	0.077
55-75 (*N* = 1,312)	0.604 (0.378-0.965)	0.035	0.535 (0.323-0.885)	0.015
65-89 (*N* = 1,176)	0.815 (0.562-1.183)	0.283	0.727 (0.484-1.092)	0.124

*Confounders: Angiotensin-converting enzyme inhibitors, Angiotensin II receptor blockers, Diuretics, Beta blockers, Calcium-channel blockers, Statins, Glucocorticoids, Age, Race, Smoking Status, Body Mass Index, Temperature at admission, Max temperature during hospitalization, O2 Saturation at admission, Minimum O2 saturation during hospitalization, Heart rate at admission, Respiratory rate, High blood pressure, Asthma, Chronic obstructive pulmonary disease, Hypertension, Diabetes Mellitus, Obstructive Sleep Apnea, Obesity, Chronic Kidney Disease, Human immunodeficient virus, Cancer, Coronary artery disease, Atrial fibrillation, Heart failure, Chronic viral hepatitis, Liver disease, Acute kidney injury, White blood cell count, Creatinine, Anion gap, Potassium, Alanine aminotransferase, Ferritin, ICU, Hospital duration*.

## Discussion

Using a racially and ethnically diverse cohort from New York City comprising 2,627 men aged 45 or older hospitalized COVID-19 patients seen between February 24 and May 31, 2020, we found that inpatient use of α_1_-blockers was significantly associated with reduced in-hospital mortality after adjusting for several confounders. In age-stratified analyses, α_1_-blocker exposure appeared more protective in the 55-75 year age group.

Drug repurposing is the process of finding new indications for drugs already in clinical use. The appeal of rapidly validating and deploying an existing drug against a deadly global pandemic is clear, especially if the drug is widely available and affordable. Dexamethasone, now a standard in COVID-19 treatment, is an example of a commonly used drug repurposed for a new indication (3). However, the saga of hydroxychloroquine, which was touted as a cure early in the pandemic but has since proven ineffective, is a cautionary tale (15). The allure of rapid drug repurposing must be balanced against rigorous scientific method.

α_1_-blockers, commonly used to treat benign prostatic hyperplasia and hypertension, have become a target for drug repurposing due to preclinical data linking α_1_-adrenergic signaling to pro-inflammatory cytokines which may contribute to dysregulated immunity and adverse outcomes in COVID-19 (1, 2, 11). These preclinical findings have been bolstered by recent retrospective clinical analyses linking α_1_-blockers with improved outcomes in hospitalized patients with both COVID-19 and non-COVID-19 respiratory infections (12, 13). In a large COVID-19 cohort drawn from the US Veterans Health Administration hospital system, outpatient α_1_-blocker exposure was associated with a relative risk reduction of 18% for in-hospital mortality compared to matched controls (13). Interestingly, the non-selective α_1_-blocker doxazosin, which inhibits all three α_1_-adrenergic receptor subtypes (α_1A_, α_1B_, α_1D_), was associated with a greater relative risk reduction (74%) than the uroselective (α_1A_, α_1D_) α_1_-blocker tamsulosin (18%).

In the present study, we found that in-hospital use of α_1_-blockers was independently associated with reduced in-hospital mortality after controlling for confounders such as demographics, comorbidities, and clinical factors such as vital signs and lab values. In age-stratified analyses, we observed that this protective effect was more pronounced in the 55-75 year age group. In contrast to the studies by Koenecke et al. and Rose et al., which defined α_1_-blocker exposure based on outpatient prescriptions only, we were able to use inpatient medication administration records to identify patients treated with α_1_-blockers during their COVID-19 hospitalization. The stronger effect seen in the inpatient exposure group than the overall group lends additional support to the hypothesis that α_1_-blockers may have a beneficial effect against COVID-19.

Our results also include tests of association between other common medication classes and COVID-19 outcomes, including beta-blockers, angiotensin-converting enzyme (ACEi) inhibitors, angiotensin II receptor blockers (ARBs), and glucocorticoids. Results pertaining to these other medications should be taken in the context of a selected cohort designed to study α_1_-blocker use and COVID-19 outcomes. That said, it is interesting to note that glucocorticoids were associated with worse COVID-19 outcomes, contrary to the results of a randomized controlled trial (3). This discrepancy may be due to indiscriminate administration of steroids early in the pandemic, as seen in other RWE studies. For example, the use of high-dose steroids was associated with higher odds of death (16). In this study, a high dose was classified by >40 mg daily of methylprednisolone equivalent dosing. For comparison, the equivalent to the RECOVERY trial dosing of 6 mg dexamethasone is 20-30 mg of methylprednisolone (16). Furthermore, corticosteroids were associated with an increased risk of death in patients younger than 60 years without inflammation on admission (17). Thus, the observed effect of steroids in this real-world study may diverge from the effect reported in randomized trials due to factors such as inconsistent dosing, steroid choice, and patient selection early in the pandemic.

Also of interest, α_1_-blocker and beta-blocker exposure were associated with opposite COVID-19 outcomes in our cohort. There is evidence to suggest that β-adrenergic signaling can promote an anti-inflammatory M2 phenotype in macrophages, in contrast to the pro-inflammatory effect of α_1_-adrenergic signaling (11, 18). Additional efforts to dissect the interactions between adrenergic signaling and the COVID-19 immune response are warranted. A prior diagnosis of asthma was associated with reduced in-hospital mortality in this analysis. While this observation deserves further scrutiny, it is conceivable that early exposure to inhaled glucocorticoids or β-adrenergic agonists may have contributed to this signal.

### Limitations

Our study has several limitations. The cohort did not include women since most α_1_-blockers were prescribed to men, most likely for benign prostatic hyperplasia. Male sex is a recognized risk factor for adverse COVID-19 outcomes, possibly due to sex-specific differences in immunity (19). Thus, these results may not extrapolate to women. We did not account for different types of α_1_-blockers, which differentially target the three α_1_-adrenergic receptor subtypes.

Importantly, a causal relationship cannot be definitively established between α_1_-blockers and improved COVID-19 outcomes in this retrospective study. Several confounders, such as older age, comorbidities, and hypoxia (an indicator of COVID-19 severity), were more common in the α_1_-blocker group. However, these adverse risk factors would be expected to bias the study result toward the null rather than inflate a protective association. Furthermore, our findings are consistent with prior data (13). Ongoing randomized clinical trials of prazosin (20) and doxazosin (21) against a placebo among hospitalized COVID-19 patients will include women and provide more definitive data on the therapeutic value of α_1_-blockers.

Finally, outpatient medication adherence cannot be evaluated from the EMR. However, inpatient medication administrations are captured by the EMR and provide a definitive record of exposures. Therefore, analyzing in-hospital medication administration is more robust.

## Conclusions

In conclusion, this retrospective study found a protective association between α_1_-blocker exposure and COVID-19 outcomes in a cohort of hospitalized men. These results augment the rationale for studying and repurposing α_1_-blockers as a COVID-19 therapeutic. Thus, we await the results of two ongoing randomized clinical trials (20, 21) to definitively assess the effectiveness of alpha-1-blockers in protecting patients against COVID-19.

## Materials and Methods

### Data Sources

This retrospective study utilized de-identified electronic medical record (EMR; Epic Systems, Verona, WI) data from five member hospitals within the Mount Sinai Health System (MSHS) in the New York City metropolitan area (MS BI Brooklyn, MS St. Luke's, The Mount Sinai Hospital, MS Queens Hospital, and MS West). De-identified EMR data were obtained via the Mount Sinai Data Warehouse (https://labs.icahn.mssm.edu/msdw/). COVID-19 was diagnosed by real-time reverse transcriptase polymerase chain reaction (RT-PCR)-based clinical tests from nasopharyngeal swab specimens. In total, we identified 8,442 MSHS patients with PCR confirmed diagnosis of COVID-19 from February 24 through May 31, 2020, during the peak of the pandemic in NYC.

We retrieved patient demographics, social history, medication history, and disease comorbidities from the EMR including age, gender, race/ethnicity, smoking status, asthma, chronic obstructive pulmonary disease (COPD), hypertension, obstructive sleep apnea, obesity, diabetes, chronic kidney disease, human immunodeficient virus (HIV) infection, cancer, coronary artery disease, atrial fibrillation, heart failure, chronic viral hepatitis, alcoholic non-alcoholic liver disease, and acute kidney injury (AKI). Patients aged ≥ 89 years were assigned an age of 89 to prevent re-identification. Medications by prescription or hospital administration captured in EMR from January 1, 2019, till May 31, 2020, were included in the medication history. We identified disease comorbidities through their corresponding ICD-10-CM codes before hospital admission and during hospitalization.

We also extracted data from each hospital encounter, including vital signs and laboratory data at the time of presentation, and medications administered during hospitalization. Vital sign and laboratory data extracted included: white blood cell count (WBC), serum creatinine, anion gap, potassium, alanine aminotransferase (ALT), body mass index (BMI), temperature, oxygen saturation, heart rate, respiratory rate, systolic blood pressure (SBP) and diastolic blood pressure (DBP).

This study was approved by the Mount Sinai institutional review board (IRB): IRB-17-01245.

### Study Design

This was a retrospective EMR-based study designed to test the independent association of α_1_-blocker exposure with in-hospital death among COVID-19 patients. We first identified 6,218 inpatients positive for COVID-19 in one of five hospital systems within the MSHS as of May 31, 2020 (Figure 1A). The majority (93%) of α-_1_-blocker users in this cohort were men aged 45 or older. Therefore, we restricted the analysis cohort to men aged 45 or older (*N* = 2,627) to limit confounding due to the associations between older age/male sex with both the exposure (α_1_-blocker usage) and the outcome (COVID-19 outcomes).

The primary endpoint was in-hospital mortality. We defined two possible outcomes for each hospitalization: in-hospital death (deceased) or discharged to home or other locations not associated with acute medical care (recovered). The duration of hospitalization was calculated from the beginning of the hospital encounter till death or discharge.

The primary predictor was α_1_-blocker exposure, which we defined as an active prescription from January 1, 2020, for an α_1_-blocker (tamsulosin, alfuzosin, silodosin, terazosin, doxazosin, and prazosin) up to and including hospitalization for COVID-19 (*N* = 436). We further defined a subset of patients (*N* = 343) with documented α_1_-blocker administration during their hospitalization, which we defined as “α_1_-blocker inpatient use” (Figure 1A).

Potential confounders in the analysis included demographic characteristics, comorbidities, baseline labs and vitals, and exposure to medications used to treat hypertension, hyperlipidemia, and inflammation. Confounders were selected *a priori* based on the literature, clinician input, and data completeness. Detailed medication names included in these categories are shown in Supplementary Table 1. Certain co-morbidities, e.g., obesity, are reported as conditions that patients carried prior to hospital admission. We required that at least 85% of patients report a value for a potential confounder for it to be included in the analysis. Some potential confounders that have been associated with severe forms of COVID-19 since the beginning of the pandemic did not meet this threshold, e.g., baseline Ferritin and LDH measures. However, we did include Ferritin values recorded at hospitalization as there was reasonable coverage (75.1%).

### Statistical Analyses

Patient characteristics were summarized as median and interquartile range (IQR) for continuous variables or mean and standard deviation (SD). We displayed categorical variables as number and percentage (%). We performed a statistical test of hypothesis for differences using the Kruskal-Wallis test or two sample *t*-test for continuous variables, and the χ^2^ test for categorical variables.

We employed multivariate logistic regression models with potential confounders to estimate the odds ratio and corresponding 95% confidence interval for COVID-19 in-hospital mortality (deceased = 1) vs. recovery (recovered = 0) associated with α_1_-blocker use. We adjusted for the following confounders, which were selected *a priori*: age, hospital stay duration, race, smoking status, BMI, temperature, O2 saturation, heart rate, respiratory rate, hypertension, asthma, COPD, obstructive sleep apnea, obesity, diabetes mellitus, chronic kidney disease, HIV, cancer, coronary artery disease, atrial fibrillation, heart failure, chronic viral hepatitis, liver disease, AKI, ICU stay, WBC, creatinine, anion gap, potassium, and ALT. We used two-tailed test to estimate the probability of event under the null hypothesis which was the in-hospital mortality rate, and we used the binomial distribution for α_1_-blocker with the prescription rate as the probability.

Statistical significance was defined as a two-sided *P*-value < 0.05, unless otherwise noted.

## Data Availability Statement

The data analyzed in this study is subject to the following licenses/restrictions: our data collection, cleaning, and quality control framework makes use of proprietary data structures and libraries, so we are not releasing or licensing this code. We provide implementation details in the methods section to allow for independent replication. Requests to access these datasets should be directed to li.li@sema4.com.

## Ethics Statement

This study was approved by the Mount Sinai Institutional Review Board (IRB): IRB-17-01245.

## Author Contributions

WO, LL, and SL conceived and designed the study. SL, ZW, and ES performed data extraction from MSHS. SL, Y-HK, and JT performed statistical analysis. TJ, MK, CB, JV, NP, ES, RP, RC, LL, and WO contributed clinical interpretation. SL, TJ, Y-HK, JT, MK, CB, JV, NP, ES, RP, RC, LL, and WO wrote and edited the paper. All authors contributed to the article and approved the submitted version.

## Funding

MK was supported by grants from the Jerome L. Greene Foundation (Discovery and Scholar Award) and the Peter and Carmen Lucia Buck Foundation.

## Conflict of Interest

SL, TJ, JT, ES, Y-HK, ZW, RC, EES, LL, and WKO were all employees of Sema4 at the time this research was conducted. Sema4 is a publicly traded, for-profit company in which the Icahn School of Medicine at Mount Sinai (ISMMS) holds equity. RC, EES, LL, and WKO receive compensation from Sema4 that includes equity in the company. In addition to their roles with Sema4, RC, EES, and WKO remain affiliated with ISSMS as part-time employees and faculty members. WKO also has consulted for AAA, Astellas, AstraZeneca, Bayer, Conjupro, Foundry, Janssen, Merck, Sanofi and TeneoBio. The JHU filed a patent application on the use of various drugs to prevent cytokine release syndromes, on which NP is listed as an inventor. JHU will not assert patent rights from this filing for treatment related to COVID-19. NP is a founder of, consultant to and holds equity in Thrive an Exact company. NP is a founder of and holds equity in Personal Genome Diagnostics. NP is an advisor to and holds equity in Cage Pharma, ManaTbio, and NeoPhore. CB is a consultant to Depuy-Synthes and Bionaut Labs. CB, MFK, and NP are also inventors on technologies unrelated or indirectly related to the work described in this article. MFK is a consultant to argenx. Licenses to these technologies are or will be associated with equity or royalty payments to the inventors, as well as to JHU. The terms of all these arrangements are being managed by JHU in accordance with its conflict-of-interest policies. JV is supported by grants from Microsoft Research and Fast Grants. Microsoft Research and Fast Grants were not involved in the study design, collection, analysis, interpretation of data, the writing of this article, or the decision to submit it for publication. The remaining authors declare that the research was conducted in the absence of any commercial or financial relationships that could be construed as a potential conflict of interest.

## Publisher's Note

All claims expressed in this article are solely those of the authors and do not necessarily represent those of their affiliated organizations, or those of the publisher, the editors and the reviewers. Any product that may be evaluated in this article, or claim that may be made by its manufacturer, is not guaranteed or endorsed by the publisher.
