# Supplementary material for: Inpatient Administration of Alpha-1-Adrenergic Receptor Blocking Agents Reduces Mortality in Male COVID-19 Patients
Source: Front Med (Lausanne). 2022 Feb 28;9:849222. doi: 10.3389/fmed.2022.849222 (PMC8919772; doi:10.3389/fmed.2022.849222)
Supplement: Supplementary file 1 [file Data_Sheet_1.docx]

**Supplementary Material**

*Table S1: Medication categories and individual medications.*

| **Medication Category** | **Medication Names** |
| --- | --- |
| Alpha-1 Blockers | Tamsulosin, Alfuzosin, Silodosin, Terazosin, Doxazosin, Prazosin |
| ACE inhibitors | Benazepril hydrochloride, Captopril, Enalapril maleate, Fosinopril sodium, Lisinopril, Moexipril, Perindopril, Quinapril hydrochloride, Ramipril, Trandolapril |
| ARBs | Azilsartan, Candesartan, Eprosartan mesylate, Irbesartan, losartan potassium, Olmesartan, Telmisartan, Valsartan |
| Diuretics | Chlorthalidone, Chlorothiazide, Hydrochlorothiazide, Indapamide, Metolazone, Amiloride hydrochloride, Spironolactone, Triamterene, Eplerenone, Furosemide, Bumetanide, Torsemide, Ethacrynic acid |
| Beta Blockers | Acebutolol, Atenolol, Betaxolol, Bisoprolol fumarate, Carteolol hydrochloride, Metoprolol, Nadolol, Nebivolol, Penbutolol sulfate, Pindolol, Propranolol hydrochloride, Solotol hydrochloride, Timolol maleate |
| Calcium-channel Blockers | Amlodipine, Bepridil, Diltiazem hydrochloride, Felodipine, Isradipine, Nicardipine, Nifedipine, Nisoldipine, Verapamil hydrochloride |
| Statin | Atorvastatin, Fluvastatin, Lovastatin, Pitavastatin, Pravastatin, Rosuvastatin, Simvastatin |
| Glucocorticoid | Methylprednisolone, Prednisone, Hydrocortisone, Dexamethasone, Fludrocortisone, Triamcinolone, Prednisolone, Betamethasone |

*Table S2: Unadjusted OR values and p-values.*

| Alpha-blocker | Deceased | | Discharged | |
| --- | --- | --- | --- | --- |
| Exposed (n) | 148 | | 288 | |
| Not exposed (n) | 629 | | 1562 | |
|  | Estimate | LCB | UCB | p-value |
| Odds Ratio | 1.276 | 1.025 | 1.588 | 0.029 |
| Alpha-blocker Inpatient | Deceased | | Discharged | |
| Exposed (n) | 112 | | 231 | |
| Not exposed (n) | 629 | | 1562 | |
|  | Estimate | LCB | UCB | p-value |
| Odds Ratio | 1.204 | 0.943 | 1.537 | 0.136 |

Abbreviations: LCB, Lower Confidence Bound; UCB, Upper Confidence Bound.
